# Comparison of Procedure-Based and Diagnosis-Based Identifications of Severe Sepsis and Disseminated Intravascular Coagulation in Administrative Data

**DOI:** 10.2188/jea.JE20150286

**Published:** 2016-10-05

**Authors:** Hayato Yamana, Hiromasa Horiguchi, Kiyohide Fushimi, Hideo Yasunaga

**Affiliations:** 1 Department of Clinical Epidemiology and Health Economics, School of Public Health, The University of Tokyo, Tokyo, Japan; 1 東京大学大学院医学系研究科公共健康医学専攻臨床疫学・経済学; 2 Department of Clinical Data Management and Research, Clinical Research Center, National Hospital Organization Headquarters, Tokyo, Japan; 2 国立病院機構本部総合研究センター診療情報分析部; 3 Department of Health Policy and Informatics, Tokyo Medical and Dental University Graduate School of Medicine, Tokyo, Japan; 3 東京医科歯科大学大学院医療政策情報学分野

**Keywords:** administrative data, procedure record, severe sepsis, disseminated intravascular coagulation, 診療情報データベース, 処置情報, 重症敗血症, 播種性血管内凝固症候群

## Abstract

**Background:**

Diagnoses recorded in administrative databases have limited utility for accurate identification of severe sepsis and disseminated intravascular coagulation (DIC). We evaluated the performance of alternative identification methods that use procedure records.

**Methods:**

We obtained data for adult patients admitted to intensive care units in three hospitals during a 1-year period. Severe sepsis and DIC were identified by three means: laboratory data, diagnoses, and procedures. Using laboratory data as a reference, the sensitivity and specificity of procedure-based methods and diagnosis-based methods were compared.

**Results:**

Of 595 intensive care unit admissions, 212 (35.6%) and 81 (13.6%) were identified as severe sepsis and DIC, respectively, using laboratory data. The sensitivity of procedure-based methods for identifying severe sepsis was 64.2%, and the specificity was 65.3%. Two diagnosis-based methods —the Angus and Martin algorithms— exhibited sensitivities of 21.7% and 14.6% and specificities of 98.7% and 99.5%, respectively, for severe sepsis. For DIC, the sensitivity of procedure-based methods was 55.6%, and the specificity was 67.1%, and the sensitivity and specificity of diagnosis-based methods were 35.8% and 98.2%, respectively.

**Conclusions:**

Procedure-based methods were more sensitive and less specific than diagnosis-based methods in identifying severe sepsis and DIC. Procedure records could improve disease identification in administrative databases.

## 抄録

**【背景】:**

診療情報データベースに記録された診断名から重症敗血症や播種性血管内凝固症候群（DIC）を正確に特定することは困難である。本研究では、入院中に実施された処置の情報を用いた新たな特定方法の性能を評価した。

**【方法】:**

1年間の観察期間中に3施設の集中治療室に入室した成人患者の情報を、国立病院機構データベースより抽出した。重症敗血症およびDICを検査値、診断名、処置情報の3通りの方法により特定し、検査値による方法の判定結果を対照として診断名および処置情報による方法の感度、特異度等を算出した。

**【結果】:**

集中治療室に入室した595人のうち、検査値により212人（35.6%）が重症敗血症、81人（13.6%）がDICと判定された。重症敗血症の特定において、処置情報による方法は感度64.2%、特異度65.3%を示した。診断名による方法は、Angus法が感度21.7%、特異度98.7%を示し、Martin法が感度14.6%、特異度99.5%を示した。DICの特定において、処置情報による方法は感度55.6%、特異度67.1%を示し、診断名による方法は感度35.8%、特異度98.2%を示した。

**【結論】:**

重症敗血症およびDICの特定において、処置情報による方法は診断名による方法と比較して高い感度を示したが特異度は低かった。処置情報の利用はデータベースにおける疾患の特定方法を改善させる可能性がある。

## INTRODUCTION

Severe sepsis and disseminated intravascular coagulation (DIC) are two critical conditions associated with high mortality.^1^^–^^4^ Sepsis is defined as a systemic inflammatory response to an infection, with the term “severe sepsis” used to describe sepsis complicated by acute organ dysfunction.^4^ DIC is characterized by the widespread activation of coagulation, which results in intravascular formation of fibrin and ultimately thrombotic occlusion of vessels.^2^^,^^3^ Scoring systems using clinical laboratory tests to diagnose DIC have been proposed and validated.^5^^–^^8^ In addition to clinical studies, large administrative databases have been used to investigate the epidemiology of severe sepsis and DIC.^9^^–^^15^

Despite the widespread use of administrative databases, there are no established methods that can accurately identify severe sepsis and DIC in databases. Validation studies have indicated that extraction of recorded diagnoses from administrative databases has low sensitivity in identifying severe sepsis.^16^^–^^18^ Further, previous reports have found substantial variability in the incidence and severity of severe sepsis across different extraction methods.^19^^–^^22^ For DIC, no studies have evaluated the validity of recorded diagnoses.

Some administrative databases record performed procedures in addition to diagnoses and patient demographics.^23^^,^^24^ Using this additional information, more accurate identification of severe sepsis and DIC may be possible. However, there have been no reports of such methods.

The aim of the present study was to develop methods that can use procedure records to identify severe sepsis and DIC in administrative databases. Using laboratory data recorded in a database as the “gold standard”, we compared the characteristics of procedure records with those of diagnoses in identifying severe sepsis and DIC.

## METHODS

### Data source

The National Hospital Organization (NHO) was established in 2004 to take over the management of the national hospitals in Japan. As of April 2015, there were 143 hospitals nationwide run by the NHO, including both general acute-care hospitals and specialized long-term-care hospitals. All NHO hospitals provide administrative claims data to the Medical Information Analysis (MIA) databank managed by the Clinical Research Center at NHO Headquarters.

In Japan, a lump-sum payment system based on the Diagnosis Procedure Combination (DPC) was introduced in acute-care hospitals nationwide in 2003.^25^ In NHO hospitals with implementation of the DPC-based payment system, the discharge abstracts and claims data used in the DPC-based payment system (DPC data) are stored in the MIA databank. As of March 2014, 52 of 142 NHO hospitals were participating in the system. Of these 52 participating hospitals, 37 were equipped with intensive care units (ICUs) and 18 were designated as tertiary emergency centers. The average number of acute-care beds in each of the 52 hospitals was 430 (range, 144–730).

In 2013, the NHO preliminarily introduced the Standardized Structured Medical Record Information Exchange (SS-MIX) standardized storage^26^ to its hospitals. The SS-MIX storage enables medical chart information, including daily laboratory data, to be recorded in a standardized manner. In this study, we collected data recorded in the SS-MIX storage of three acute-care NHO hospitals that had implemented the DPC-based payment system. The average number of acute-care beds in these hospitals was 430; all had ICUs, and one was a tertiary emergency center. We also used the MIA databank to obtain the DPC data provided from the three hospitals.

### Study population

Using patient hospitalization and in-hospital transfer information recorded in the SS-MIX storage, we identified patients hospitalized and discharged between April 1, 2013, and March 31, 2014, with ICU admission in the same period. The exclusion criteria were: age <18 years; postsurgical ICU admission (patients with surgery under general anesthesia on the day of ICU admission); admission-precipitating diagnosis^24^ of ischemic heart disease (International Classification of Diseases Tenth Revision [ICD-10] codes I20–I25); and lack of laboratory data. For patients with multiple nonsurgical ICU admissions within a single hospitalization, we included the first ICU admission.

### Variables

The following patient information was extracted from the DPC discharge abstract data: age; sex; diagnoses (up to 12, including main diagnosis, admission-precipitating diagnosis, comorbidities present at time of admission, and conditions arising after admission); consciousness rating, using the Japan Coma Scale (JCS)^27^^,^^28^; and discharge status. From the DPC claims data, we extracted daily performance of diagnostic and therapeutic procedures, along with blood transfusions, use of antibiotics, and use of catecholamines (epinephrine, norepinephrine, dopamine, or dobutamine) and vasopressin. The following daily laboratory data from the SS-MIX storage were examined: white blood cell (WBC) count, platelet count, prothrombin time-international normalized ratio (PT-INR), creatinine, and total bilirubin. For patients in whom the same examination was repeated within a single day, the most extreme values were used.

To identify infection and organ failure using recorded diagnoses, we used two coding algorithms, the Angus and Martin methods, both of which were adapted to ICD-10 codes by Wilhelms et al.^20^ DIC was identified using ICD-10 codes D65, D68.9, and D69.9. All 12 diagnoses in the DPC discharge abstract data were used in the identification.

In line with published guidelines,^29^^,^^30^ we defined “laboratory data-based” infection and organ dysfunction as those meeting the following criteria on any day during hospitalization: infection (WBC count >12 000 or <4000 µL^−1^), hematologic dysfunction (PT-INR >1.5 or platelet count <100 000 µL^−1^), renal dysfunction (creatinine >2.0 mg/dL), and hepatic dysfunction (total bilirubin >2.0 mg/dL). These values corresponded to a Sepsis-related Organ Failure Assessment (SOFA) score^31^ of ≥2. Other organ dysfunctions (cardiovascular, respiratory, and neurological dysfunctions) were undeterminable from the laboratory data and were not evaluated in this study.

We defined “procedure-based” infection, organ dysfunction, and systemic inflammatory response syndrome (SIRS) as use of the following on any day during hospitalization: intravenous antibiotics (infection); catecholamine or vasopressin (cardiovascular dysfunction); mechanical ventilation (respiratory dysfunction); heparin or transfusion of fresh-frozen plasma or platelets (hematologic dysfunction); hemodialysis or continuous hemodiafiltration (renal dysfunction); hemoadsorption or plasma exchange (hepatic dysfunction); and the combination of intravenous antibiotic, heart rate/respiration monitoring, and oxygen administration or mechanical ventilation (SIRS).

Using the laboratory data of platelet count, prothrombin time, and fibrin degradation products, we also calculated the daily revised Japanese Association for Acute Medicine (JAAM) DIC scores^7^^,^^8^ and defined laboratory data-based DIC as a JAAM DIC score of ≥4 on any day during hospitalization. As physiological information was unavailable in the database, SIRS criteria in the JAAM DIC score were undeterminable. Therefore, we also defined laboratory data-based possible DIC as a maximum JAAM DIC score of 3 within a single hospitalization. Procedure-based DIC was defined as procedure-based SIRS plus hematologic dysfunction.

### Statistical analysis

We defined laboratory data-based severe sepsis as the presence of laboratory data-based hematologic, renal, or hepatic dysfunction in addition to laboratory data-based infection. Diagnosis- and procedure-based severe sepsis was identified in a similar manner. For diagnosis- and procedure-based methods, we also identified severe sepsis using all organ failures. Using the laboratory data-based method as a reference (gold standard), we calculated the sensitivity, specificity, positive predictive value (PPV), and negative predictive value (NPV) of the other two methods of identifying infection, organ dysfunction, severe sepsis (three organ failures), and DIC. Among the laboratory data-positive or laboratory data-negative severe sepsis patients, the in-hospital mortality rates were compared between diagnosis-positive and diagnosis-negative patients using Fisher’s exact test. The mortality rates of procedure-positive and procedure-negative patients were also compared. The same comparisons were conducted for DIC patients. A two-sided *P*-value of <0.05 was considered significant. In addition, we compared the identification of severe sepsis using all organ failures between diagnosis-based and procedure-based methods. The kappa coefficient was used to evaluate the agreement between the two methods. All statistical analyses were conducted using IBM SPSS for Windows, version 21.0 (IBM Corp., Armonk, NY, USA).

### Standard protocol approvals, registrations, and patient consent

The study protocol was approved by the Central Ethical Review Board of NHO, which deemed written informed consent from participants unnecessary.

## RESULTS

We identified 1528 adult ICU admissions to the three hospitals in the 1-year study period. There were 743 postsurgical ICU admissions and 168 admissions for ischemic heart diseases. We further excluded two patients with missing laboratory data and 20 patients with second or later medical ICU admissions within a single hospitalization, leaving 595 independent hospitalizations for analysis. The demographic characteristics of the ICU-admitted patients are presented in Table 1. There were 113 in-hospital deaths (19.0%), including 56 patients who died in the ICU (9.4%).

**Table 1.** Patient demographics (*n* = 595)

Male sex	360	(60.5)
Mean (SD) age, years	70.7	(15.1)
Order of ICU admission within hospitalization		
First	570	(95.8)
Second (after postsurgical ICU stay)	25	(4.2)
Median (IQR) days of ICU admission	1	(1, 4)
Median (IQR) length of ICU stay, days	3	(2, 5)
Median (IQR) length of hospitalization, days	23	(12, 39)
JCS score		
0	442	(74.3)
1–3	79	(13.3)
10–30	26	(4.4)
100–300	48	(8.1)
Main diagnosis^a^		
Infectious diseases (A, B)	19	(3.2)
Neoplasms (C, D)	66	(11.1)
Circulatory (I)	264	(44.4)
Respiratory (J)	43	(7.2)
Digestive (K)	66	(11.1)
External causes (S, T)	75	(12.6)
Other	62	(10.4)
Outcomes		
In-hospital death	113	(19.0)
In-ICU death	56	(9.4)

ICU, intensive care unit; JCS, Japan Coma Scale.

Data are presented as *n* (%), unless otherwise noted.

^a^Letters represent International Classification of Diseases Tenth Revision codes.

The results for identification using recorded diagnoses are presented in Table 2. Severe sepsis was diagnosed in 77 patients (12.9%) and 43 patients (7.2%) using the Angus and Martin methods, respectively. When limiting organ dysfunctions to hematologic, renal, and hepatic dysfunctions, severe sepsis was indicated in 51 patients (8.6%) using the Angus methods and 33 patients (5.5%) using the Martin method. DIC was identified in 38 patients (6.4%).

**Table 2.** Identification using recorded diagnoses (*n* = 595)

	*n* (%) of patients identified			

	Angus		Martin	
Infection	188	(31.6)	64	(10.8)
Organ failures				
Cardiovascular	93	(15.6)	93	(15.6)
Respiratory	1	(0.2)	52	(8.7)
Hematologic	51	(8.6)	52	(8.7)
Renal	25	(4.2)	34	(5.7)
Hepatic	14	(2.4)	14	(2.4)
Neurologic	4	(0.7)	18	(3.0)
Metabolic	N/A		3	(0.5)
Severe sepsis (all organ failure)	77	(12.9)	43	(7.2)
Severe sepsis (three organ failures^a^)	51	(8.6)	33	(5.5)

^a^Hematologic, renal, or hepatic failure.

The results for identification using laboratory data or procedures are presented in Table 3. Using laboratory data, 212 (35.6%), 81 (13.6%), and 111 (18.7%) patients were identified as having severe sepsis (by three organ failures), DIC, and possible DIC, respectively. Using procedures, severe sepsis was identified in 309 patients (51.9%). When limited to three organ failures, 269 patients (45.2%) were identified. SIRS and DIC was identified in 290 patients (48.7%) and 214 patients (36.0%), respectively.

**Table 3.** Identification using laboratory data or procedures (*n* = 595)

	*n* (%) meeting laboratory data criteria		*n* (%) meeting procedure criteria	
Infection	371	(62.4)	364	(61.2)
Organ failures				
Cardiovascular	N/A		221	(37.1)
Respiratory	N/A		205	(34.5)
Hematologic	160	(26.9)	327	(55.0)
Renal	152	(25.5)	69	(11.6)
Hepatic	91	(15.3)	5	(0.8)
SIRS	N/A		290	(48.7)
Severe sepsis (five organ failures)	N/A		309	(51.9)
Severe sepsis (three organ failures^a^)	212	(35.6)	269	(45.2)
DIC	81	(13.6)	214	(36.0)
Possible DIC	111	(18.7)	N/A	

DIC, disseminated intravascular coagulation; N/A, not applicable; SIRS, systemic inflammatory response syndrome.

^a^Hematologic, renal, or hepatic failure.

Comparisons of the diagnosis- and procedure-based identifications with the laboratory data-based identification are shown in Table 4. Compared with diagnosis-based methods, procedure-based methods had higher sensitivity and lower specificity in identifying severe sepsis and DIC. PPVs were higher for diagnosis-based methods in severe sepsis and DIC patients. NPV was higher for procedure-based methods in severe sepsis patients, and similar for the two methods in DIC patients.

**Table 4.** Comparisons between diagnosis-, procedure-, and laboratory-data-based identifications

		Laboratory data-positive		Laboratory data-negative		Predictive value			Laboratory data-positive		Laboratory data-negative		Predictive value
					
		*n*	(%)	*n*	(%)	%			*n*	(%)	*n*	(%)	%
	
Infection							Organ failure (hematologic)						
Angus	+	150	(40.4)	38	(17.0)	79.8	Angus	+	40	(25.0)	11	(2.5)	78.4
	−	221	(59.6)	186	(83.0)	45.7		−	120	(75.0)	424	(97.5)	77.9
Martin	+	61	(16.4)	3	(1.3)	95.3	Martin	+	40	(25.0)	12	(2.8)	76.9
	−	310	(83.6)	221	(98.7)	41.6		−	120	(75.0)	423	(97.2)	77.9
Procedure	+	267	(72.0)	97	(43.3)	73.4	Procedure	+	110	(68.8)	217	(49.9)	33.6
	−	104	(28.0)	127	(56.7)	55.0		−	50	(31.3)	218	(50.1)	81.3
Total		371		224			Total		160		435		
Severe sepsis							Organ failure (renal)						
Angus	+	46	(21.7)	5	(1.3)	90.2	Angus	+	20	(13.2)	5	(1.1)	80.0
	−	166	(78.3)	378	(98.7)	69.5		−	132	(86.8)	438	(98.9)	76.8
Martin	+	31	(14.6)	2	(0.5)	93.9	Martin	+	29	(19.1)	5	(1.1)	85.3
	−	181	(85.4)	381	(99.5)	67.8		−	123	(80.9)	438	(98.9)	78.1
Procedure	+	136	(64.2)	133	(34.7)	50.6	Procedure	+	60	(39.5)	9	(2.0)	87.0
	−	76	(35.8)	250	(65.3)	76.7		−	92	(60.5)	434	(98.0)	82.5
Total		212		383			Total		152		443		
DIC							Organ failure (hepatic)						
Diagnosis	+	29	(35.8)	9	(1.8)	76.3	Angus	+	9	(9.9)	5	(1.0)	64.3
	−	52	(64.2)	505	(98.2)	90.7		−	82	(90.1)	499	(99.0)	85.9
Procedure	+	45	(55.6)	169	(32.9)	21.0	Martin	+	9	(9.9)	5	(1.0)	64.3
	−	36	(44.4)	345	(67.1)	90.6		−	82	(90.1)	499	(99.0)	85.9
Total		81		514			Procedure	+	2	(2.2)	3	(0.6)	40.0
								−	89	(97.8)	501	(99.4)	84.9
							Total		91		504		

DIC, disseminated intravascular coagulation.

The mortality rates are presented in Table 5. Among patients identified as having severe sepsis using laboratory data, there was a significant difference in the mortality rates for Angus-positive and Angus-negative patients. Although not significant, Martin-positive patients had a higher mortality rate than Martin-negative patients. In laboratory data-identified DIC patients, there was no significant difference in the mortality rates between diagnosis-positive and diagnosis-negative patients. In both laboratory data-identified severe sepsis and laboratory data-identified DIC patients, there were no significant differences between the mortality rates of procedure-positive and procedure-negative patients.

**Table 5.** Comparisons of mortality rates

		Laboratory data-positive			Laboratory data-negative			All		
		
		Mortality rate, % (*n*)		*P*	Mortality rate, % (*n*)		*P*	Mortality rate, % (*n*)		*P*
Severe sepsis										
Angus	+	56.5	(26/46)	0.002	20.0	(1/5)	0.391	52.9	(27/51)	<0.001
	−	30.7	(51/166)		9.3	(35/378)		15.8	(86/544)	
Martin	+	48.4	(15/31)	0.158	50.0	(1/2)	0.179	48.5	(16/33)	<0.001
	−	34.3	(62/181)		9.2	(35/381)		17.3	(97/465)	
Procedure	+	38.2	(52/136)	0.460	6.0	(8/133)	0.140	22.3	(60/269)	0.074
	−	32.9	(25/76)		11.2	(28/250)		16.3	(53/326)	
Total		36.3	(77/212)		9.4	(36/383)		19.0	(113/595)	
DIC										
Diagnosis	+	62.1	(18/29)	0.485	22.2	(2/9)	0.339	52.6	(20/38)	<0.001
	−	51.9	(27/52)		13.1	(66/505)		16.7	(93/557)	
Procedure	+	53.3	(24/45)	0.822	15.4	(26/169)	0.333	23.4	(50/214)	0.0497
	−	58.3	(21/36)		12.2	(42/345)		16.5	(63/381)	
Total		55.6	(45/81)		13.2	(68/514)		19.0	(113/595)	

DIC, disseminated intravascular coagulation.

Among the patients identified as having severe sepsis by diagnoses or procedures using all organ failures, the mortality rates were 42.9% (33/77) for Angus-identified patients, 41.9% (18/43) for Martin-identified patients, and 22.7% (70/309) for procedure-identified patients. Comparisons of the diagnosis- and procedure-based identifications of severe sepsis patients using all organ failures are presented in Table 6. The kappa coefficients for agreement between diagnosis- and procedure-based identifications were 0.177 (95% CI, 0.126–0.227) for the Angus method and 0.076 (95% CI, 0.037–0.115) for the Martin method.

**Table 6.** Comparisons between diagnosis- and procedure-based identifications

Diagnosis-based identification	Procedure-based identification		

	+	−	Total
Angus			
+	67	10	77
−	242	276	518
Total	309	286	595
Martin			
+	34	9	43
−	275	277	552
Total	309	286	595

## DISCUSSION

There are several limitations to the identification of severe sepsis and DIC in administrative data using recorded diagnoses. Therefore, we developed methods to identify severe sepsis and DIC using procedure records. Using laboratory data-based identifications as a reference, procedure-based methods had higher sensitivity and lower specificity than diagnosis-based methods.

Previous studies have used clinical diagnoses of severe sepsis as a reference to examine the validity of diagnoses recorded in administrative data.^16^^–^^18^ The present study differs from these studies in that we used recorded laboratory data as a reference. Our results should be compared with those of the previous studies with caution, because the cohorts of severe sepsis and DIC patients in our study may not be as robust as those based on clinical diagnoses. For example, the laboratory data-defined organ dysfunctions in the present study may be the results of chronic conditions and not acute consequences of infection. In addition, the infection and organ dysfunction did not necessarily need to occur at the same time to be considered laboratory data-based severe sepsis. Also, laboratory data may not have been obtained using the same protocol across hospitals. Nevertheless, the high mortality rate (36.3%) of laboratory data-based severe sepsis patients is similar to rates reported for clinically-defined severe sepsis patients.^17^^,^^32^^,^^33^ This suggests that laboratory data-based identification can serve as a valid reference.

In the present study, diagnosis-based identification of severe sepsis patients was conducted using the Angus and Martin algorithms, two widely used methods. A previous validation study of the two methods showed sensitivities of 50.3% and 16.8% for the Angus and Martin methods, respectively.^18^ Another validation study of the Angus method presented a sensitivity of 47.2%.^17^ The results of the present study cannot be simply compared with these results, because the sensitivity in our study was calculated for patients with hematologic, renal, or hepatic dysfunctions only. However, the sensitivities of recorded diagnoses were also low in the present study (21.7% for the Angus method and 14.6% for the Martin method). The sensitivities for hematologic, renal, and hepatic failures were about 10%–20%. Considering that 37.1% of patients were on vasopressors and 34.5% required mechanical ventilation, the rates of cardiovascular and respiratory dysfunctions also appeared to be low. These results illustrate the limitations of the diagnosis-based identification of severe sepsis. Up to 12 diagnoses are recordable in the DPC discharge abstract data, but only four diagnoses can be recorded as comorbidities present on admission, and four can be recorded as conditions arising after admission.^24^ These limits could have lowered the sensitivities in the present study. The sensitivity for DIC diagnosis was slightly higher (35.8%), yet more than half of the DIC patients were not documented.

Among the laboratory data-identified severe sepsis patients, the mortality rate was higher in patients with diagnosis-identified severe sepsis than in those who were not identified by their diagnoses. A previous validation study also reported higher mortality rates among severe sepsis patients in whom diagnoses were recorded, suggesting that more severe patients were more likely to have documented diagnoses.^17^ Administrative databases are widely used for epidemiology studies of severe sepsis. However, this bias should be kept in mind when using diagnosis-based extraction of severe sepsis patients. In DIC patients, the difference in mortality between patients with and without documentation was relatively small. Thus, the diagnosis-based selection of DIC patients may be less biased.

Among ICU-admitted patients, 12.9% were identified as having severe sepsis using the Angus algorithm and 7.2% were identified using the Martin algorithm. The mortality rates for the Angus-positive and Martin-positive patients were 42.9% and 41.9%, respectively. The differences in the incidence and mortality rates of patients identified by the two methods were smaller than those presented in previous studies, which reported that the Angus method derived approximately three times the incidence and two-thirds the mortality rate compared with the Martin method.^19^^,^^20^^,^^22^ The differences between the two methods have been attributed to the broader selection criteria of the Angus method for identifying infection.^22^ This was also seen in our study, as 31.6% of patients were positive for infection based on the Angus criteria, while only 10.8% fulfilled the Martin criteria for infection. However, there were also differences in the numbers of patients identified as having organ failure, and fewer patients were identified by the Angus method. Only one of 595 patients had a diagnosis of respiratory failure recorded using the Angus method, despite the fact that 205 patients were on mechanical ventilation. The Angus method relies solely on ICD-10 code Z99.1 (dependence on respirator),^20^ and it is possible that this diagnosis was not recognized by physicians. Also, for other organ dysfunctions, less severely ill patients may not have been selected by the Angus method, thereby decreasing the incidence and increasing the mortality rate. The limits to the numbers of recordable diagnoses in the DPC database could have exacerbated the under-recording of diagnoses. In addition, this study was conducted in three hospitals and we only included patients with ICU admission, which could have produced a relatively homogeneous population of patients and decreased the differences between the two methods.

In the present study, procedure-based identification of severe sepsis and DIC was examined as a possible alternative to diagnosis-based identification. The sensitivity of procedure-based methods for identifying severe sepsis (three organ failures) was 64.2%, which was higher than that of diagnosis-based methods. However, the specificity (65.3%) was lower than that of diagnosis-based methods, which had a specificity of about 99%. The PPV was 50.6% under the relatively high prevalence of severe sepsis. Likewise, the sensitivity and specificity for identifying DIC were both moderate (55.6% and 67.1%, respectively), and the PPV was as low as 21.0%. Since PPV is dependent on prevalence, PPV would be expected to be lower in populations with lower prevalence of severe sepsis or DIC, limiting its use for correctly identifying severe sepsis or DIC. Overall, the procedure-based method using all organ failures identified 309 patients as having severe sepsis, of whom 70 (22.7%) died. DIC was identified in 214 patients, of whom 50 (23.4%) died. Compared with diagnosis-based methods, the procedure-based methods may have included less severe patients. When applying the procedure-based methods to research using administrative data, this difference in severity of the identified patients should be noted. The procedures we examined were relatively commonly used and not necessarily specific to severe sepsis or DIC patients. The identification of more specific procedures could increase the usability of procedure-based methods. Further studies using clinically-defined severe sepsis and DIC patients that closely examine the performed procedures are required to improve our methods.

Several limitations of this study need to be considered. First, as previously mentioned, the gold standard used in the study was recorded laboratory data, and not clinically-defined conditions. Second, the study was conducted using data for ICU-admitted patients from three hospitals. The patterns for conducting laboratory tests and procedures and for coding of diagnoses may be different in other institutions. In addition, the generalizability of the results to patients treated outside of ICUs remains unclear.

Different administrative databases have different population coverages and amounts of stored information per patient. In Japan, the nationwide DPC database collects DPC data from approximately 1000 participating hospitals. With data for approximately 7 million admissions annually, representing 50% of all acute-care admissions in the country, this database is an ideal source for population-based epidemiological studies. As the DPC database stores precise claims data in addition to diagnoses, procedure and medication records from the claims data could serve to identify diseases more accurately. This study presented examples of such methods. The NHO is currently expanding the introduction of the SS-MIX standardized storage to its hospitals to build a database, and the number of participating hospitals is planned to reach 31 by 2016. Although the coverage is small compared with the current MIA databank and the DPC database, the amount of added information per patient is large. Using this additional information, more accurate identification of diseases or conditions may be possible. Future studies that develop and validate methods to identify diseases in databases should use the information available in each database to the greatest extent possible to develop the best method.

## ONLINE ONLY MATERIAL

Abstract in Japanese.
