# Supplementary material for: Comparison of Procedure-Based and Diagnosis-Based Identifications of Severe Sepsis and Disseminated Intravascular Coagulation in Administrative Data
Source: J Epidemiol. 2016 Oct 5;26(10):530–7. doi: 10.2188/jea.JE20150286 (PMC5037250; doi:10.2188/jea.JE20150286)
Supplement: Abstract in Japanese. [file je-26-530-s001.pdf]

診療情報データベースにおける重症敗血症および播種性血管内凝固症候群の特定方法の比較

山名隼人[1,2]、堀口裕正[2]、伏見清秀[2,3]、康永秀生[1]

1　東京大学大学院医学系研究科公共健康医学専攻臨床疫学・経済学

2　国立病院機構本部総合研究センター診療情報分析部

3　東京医科歯科大学大学院医療政策情報学分野

抄録

【背景】

診療情報データベースに記録された診断名から重症敗血症や播種性血管内凝固症候群（DIC）を正確に特定することは困難である。本研究では、入院中に実施された処置の情報を用いた新たな特定方法の性能を評価した。

【方法】

1 年間の観察期間中に 3 施設の集中治療室に入室した成人患者の情報を、国立病院機構データベースより抽出した。重症敗血症および DIC を検査値、診断名、処置情報の 3 通りの方法により特定し、検査値による方法の判定結果を対照として診断名および処置情報による方法の感度、特異度等を算出した。

【結果】

集中治療室に入室した 595 人のうち、検査値により 212 人（35.6%）が重症敗血症、81 人（13.6%）が DIC と判定された。重症敗血症の特定において、処置情報による方法は感度 64.2%、特異度 65.3%を示した。診断名による方法は、Angus 法が感度 21.7%、特異度 98.7%を示し、Martin 法が感度 14.6%、特異度 99.5%を示した。DIC の特定において、処置情報による方法は感度 55.6%、特異度 67.1%を示し、診断名による方法は感度 35.8%、特異度 98.2%を示した。

【結論】

重症敗血症および DIC の特定において、処置情報による方法は診断名による方法と比較して高い感度を示したが特異度は低かった。処置情報の利用はデータベースにおける疾患の特定方法を改善させる可能性がある。

キーワード：診療情報データベース、処置情報、重症敗血症、播種性血管内凝固症候群
